# Stabilizing BiVO_4_ Photoanode in Bicarbonate Electrolyte for Efficient Photoelectrocatalytic Alcohol Oxidation

**DOI:** 10.3390/molecules29071554

**Published:** 2024-03-30

**Authors:** Haorui Gong, Sai An, Weilong Qin, Yongbo Kuang, Deyu Liu

**Affiliations:** 1 School of Materials Science and Chemical Engineering, Ningbo University, Ningbo 315211, China; gonghaorui@nimte.ac.cn (H.G.); ansai@nimte.ac.cn (S.A.); 2 Key Laboratory of Advanced Fuel Cells and Electrolyzers Technology of Zhejiang Province, Ningbo Institute of Materials Technology and Engineering, Chinese Academy of Sciences, Ningbo 315201, China; qinweilong@nimte.ac.cn; 3 Center of Materials Science and Optoelectronics Engineering, University of Chinese Academy of Sciences, Beijing 100000, China

**Keywords:** bismuth vanadate, benzyl alcohol, bicarbonate, non-aqueous medium, photostability

## Abstract

In order to expand the application of bismuth vanadate (BiVO_4_) to the field of photoelectrochemistry, researchers have explored the potential of BiVO_4_ in catalyzing or degrading organic substances, potentially presenting a green and eco-friendly solution. A study was conducted to investigate the impact of electrolytes on the photocatalysis of benzyl alcohol by BiVO_4_. The research discovered that, in an acetonitrile electrolyte (pH 9) with sodium bicarbonate, BiVO_4_ catalyzed benzyl alcohol by introducing saturated V^5+^. This innovation addressed the issue of benzyl alcohol being susceptible to catalysis in an alkaline setting, as V^5+^ was prone to dissolution in pH 9 on BiVO_4_. The concern of the photocorrosion of BiVO_4_ was mitigated through two approaches. Firstly, the incorporation of a non-aqueous medium inhibited the formation of active material intermediates, reducing the susceptibility of the electrode surface to photocorrosion. Secondly, the presence of saturated V^5+^ further deterred the leaching of V^5+^. Concurrently, the production of carbonate radicals by bicarbonate played a vital role in catalyzing benzyl alcohol. The results show that, in this system, BiVO_4_ has the potential to oxidize benzyl alcohol by photocatalysis.

## 1. Introduction

BiVO_4_ (BVO) has gained considerable attention in photoelectrochemistry (PEC) studies with its decent bandgap of 2.3–2.4 eV, as well as its excellent light absorption capabilities, enabling the conversion of light energy into chemical energy [1,2]. It demonstrates a high catalytic activity and photoelectric conversion efficiency in photocatalytic reactions [3,4].

Recent years have witnessed significant advancements in the utilization of BVO beyond its conventional role in PEC water splitting, particularly in the realm of organic synthesis [5,6,7]. Numerous electrochemical studies have demonstrated that, while alcohol oxidation is indeed thermodynamically easier than an oxygen evolution reaction (OER), it may not necessarily be kinetically favorable [8]. Hence, the key challenge lies in identifying electrochemical interfaces with appropriate catalytic properties. On the other hand, alcohols, a pivotal category in organic chemistry, encompass a diverse range of compounds, with their properties predominantly governed by hydroxyl groups [9]. The distinctive structure and robust adsorption characteristics of aromatic alcohols bestow them with a unique standing in mechanism research [10]. Aromatic alcohols, characterized by a benzene ring and a hydroxyl group, possess a unique structure that renders the hydroxyl groups within the molecule more susceptible to oxidation during chemical reactions [11]. Moreover, aromatic alcohols exhibit heightened activity across various reaction systems [12,13,14]. Hence, employing BVO to catalyze aromatic alcohols not only embodies environmental friendliness due to its low toxicity but also yields products of greater value compared to the original BVO-catalyzed water products [15,16,17].

In recent years, great progress has been made in the utilization of BVO, which goes beyond its conventional role in the water decomposition of PEC, especially in the field of organic synthesis. In contrast, significant advancements have been made in the development of photoelectrochemical catalytic structures utilizing alternative catalytic sites. Luo et al. showed that active oxygen intermediates are produced during the semi-reaction of water oxidation on the photoanode, which can generate oxygen molecules through the single-electron process, and also participate in the conversion of aromatic alcohols to aldehydes [18]. They coated BVO with double hydroxides and graphene, allowing the oxidation of benzyl alcohol (BA) to produce highly selective benzaldehyde (BAD). However, photocorrosion still limited the activity of PEC. In order to improve the performance and stability of PEC, Li et al. reported that the use of acetonitrile (MeCN) instead of water can inhibit the corrosion of BVO in water when BVO photooxidizes organic matter, providing evidence of successful oxidation in non-aqueous media. However, the performance of BVO in organic media still needs to be improved [6]. In their work on BVO photocorrosion, Lee et al. proposed that the dissolution of V^5+^ is the main cause of BVO dissolution, and the use of saturated V^5+^ electrolyte can inhibit the dissolution of BVO [19]. Yang et al. proposed the role of electrolytes in their study of the BVO oxidation of organic matter [20,21]. Carbonate free radicals are produced in the electrolyte containing bicarbonate, and HCO_3_^−^ can be used as an ideal selective oxidation medium [22,23]. Most BVO works in phosphoric acid or boric acid buffers. Although the effectiveness of bicarbonate roots has been demonstrated in the literature, the interaction of such reaction environments with the semiconductor–electrolyte solution interface remains unclear [24,25,26]. In particular, the stability of BVO in this system is poor, exhibiting fast decay within several hours [27,28]. This makes it necessary to study the evolution of the interface under working conditions.

The interfacial evolution and interaction of BVO during the oxidation of organic matter in a bicarbonate system were systematically studied. By gaining a comprehensive understanding of the interface, we optimized the reaction conditions to enhance the efficiency and stability of the photoelectrode. To combat photocorrosion, experiments and refinements were conducted based on the previous work’s reaction system [6,18,19,20]. We introduced a non-aqueous system containing bicarbonate as the electrolyte, and supplemented with saturated V^5+^. This innovative approach of incorporating a non-aqueous system and saturated V^5+^ into the electrolyte concurrently mitigates issues stemming from BVO photocorrosion. Bicarbonate acts as a mediator in the oxidation of BA to BAD, curbing the generation of by-products, and thereby affirming bicarbonate’s significant enhancement of the photocatalytic activity in BA oxidation.

## 2. Results

### 2.1. Structure of a Typical BVO

**Figure 1:**
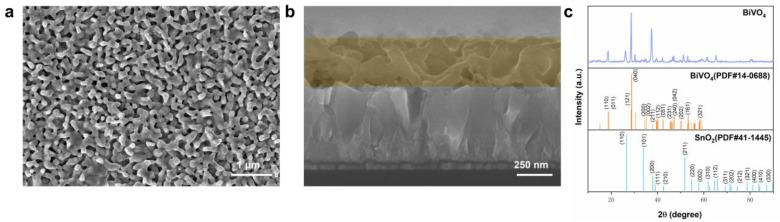
(**a**) SEM of BVO surface, (**b**) SEM of BVO cross-section, (**c**) XRD of BVO.

All the BVO utilized in the experiment was bare BVO prepared via electrodeposition. The BVO structure produced through this method exhibits a superior charge separation efficiency and enhanced control over the film thickness, crucial factors determining the photoelectrocatalytic efficiency [2,3,4]. The morphology of BVO was observed by scanning electron microscopy (SEM), and the uniform coral-striated film, as shown in Figure 1a, was observed. The yellow region in Figure 1b represents a cross-section of the BVO film with a thickness falling within the typical range, with individual variances having been deemed negligible. Notably, the PEC of BVO in a 0.5 M H_3_BO_3_ buffer (pH 9) containing sodium sulfite (Figure S1) showcased no significant deviation. The crystal structure of BVO film was further observed by X-ray Diffraction (XRD). As shown in Figure 1c, the Joint Committee on Powder Diffraction Standards (JCPDS) No. 14-0688 [29], corresponding to monolinic schewolite bismuth vanadate powder diffraction standards (FTO), was compared with SnO_2_ standard card No. 41-1445 [30]. According to the previous literature, when the diffraction peak angle 2θ = 18.67°, 28.94°, 30.54°, 34.44°, 35.22°, 39.78°, 42.46°, 46.71°, 47.30°, 50.31°, 53.24°, or 58.53°, the typical structure of BVO obtained by electrodeposition is more consistent with the diffraction standard of a monoclinic scheelite structure [31].

### 2.2. Introduction of Saturated V^5+^ for Improved Stability

**Figure 2:**
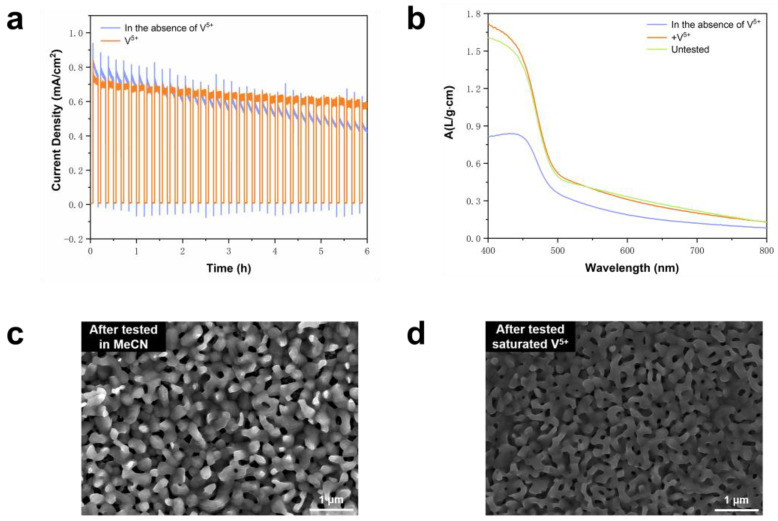
(**a**) BVO in MeCN electrolyte (pH 9) containing 0.5 M NaHCO_3_, adding saturated V^5+^ and no V^5+^ at 0.5 V vs RHE 6 h CA, (**b**) BVO UV-vis, (**c**) SEM of BVO after a 6 h test in a MeCN electrolyte (pH 9) containing 0.5 M NaHCO_3_, and (**d**) SEM of BVO after 6 h testing with saturated V^5+^ in a MeCN electrolyte (pH 9) containing 0.5 M NaHCO_3_.

BVO conducts the oxidation of BA in an MeCN electrolyte containing 0.5 M NaHCO_3_, with the inclusion of saturated V^5+^ having no discernible impact on the charge transfer between BVO holes and the electrolyte interface (Figure S2). The concentration of BA substrate added in all tests was 10 mM. Figure 2a depicts the results of the chronoamperometry (CA) test on the BVO after 6 h of irradiation under applied potential in the presence of saturated V^5+^ and in the absence of an electrolyte. Notably, the light stability examination of BVO following 6 h of testing in the two different electrolytes revealed that there is no apparent decline upon the addition of V^5+^, while a downward trend is observed in BVO stability after 2 h without the inclusion of V^5+^. As shown in Figure 2b, ultraviolet and visible spectrophotometry (UV-vis) tests were conducted on the two samples after 6 h of exposure to the applied potential test, with comparisons drawn to untested BVO. The absorption spectrum outcomes indicate that the photoanode strength of the samples tested in the V^5+^-saturated electrolyte remains consistent within the 400–800 nm range, whereas a significant reduction in BVO strength was observed in the samples tested without V^5+^. BVO tested in electrolyte containing V^5+^ has a higher absorption capacity of visible light, indicating a high substance concentration in the sample, which is due to the introduction of V^5+^ effectively inhibiting vanadium leaching from the BVO, thus inhibiting photocorrosion. Also, visually looking at the BVO sample, the BVO tested in the V^5+^-saturated electrolyte appeared to have a thicker film compared to the untested BVO (Figure S3). Figure 2c,d further confirms this observation, showing an electron microscopy morphology analysis. The BVO morphology following testing in the V^5+^-saturated electrolyte indicates minimal dissolution, while more pronounced corrosion is observed in the samples tested without the addition of V^5+^. Analysis of the High-Performance Liquid Chromatography (HPLC) results presented in Table S1 reveals that the inclusion of V^5+^ does not compromise the Faraday efficiency (FE) of BAD generation. Additionally, we conducted reactions (Figure S4) on a substantially larger 9 cm^2^ area of BVO within the same system utilizing a flow cell, achieving complete oxidation of all of the BA after 48 h, showcasing the potential for an increased catalytic yield.

### 2.3. BVO Oxidizes Benzyl Alcohol in Different Electrolytes

**Figure 3:**
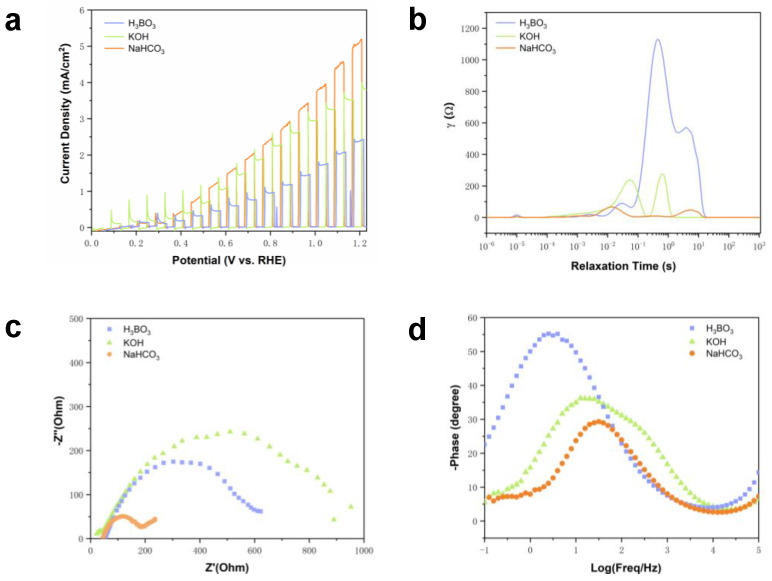
(**a**) BVO test results of LSV in 0.1 M KOH, 0.5 M H_3_BO_3_, and MeCN containing 0.5 M NaHCO_3_; (**b**) BVO test EIS at 0.6 V vs. RHE in 0.1 M KOH, 0.5 M H_3_BO_3_, and MeCN containing 0.5 M NaHCO_3_; (**c**) BVO test Nyquist plot at 0.6 V vs. RHE in 0.1 M KOH, 0.5 M H_3_BO_3_, and MeCN containing 0.5 M NaHCO_3_; (**d**) BVO test bode plot at 0.6 V vs. RHE in 0.1 M KOH, 0.5 M H_3_BO_3_, and MeCN containing 0.5 M NaHCO_3_.

We conducted a comparative analysis of typical electrolyte solutions in the BA oxidation experiment, substituting NaHCO_3_ MeCN solution with H_3_BO_3_ aqueous solution and KOH aqueous solution as electrolytes. Figure 3a illustrates the linear sweep voltammetry (LSV) results for BVO in a 0.5 M H_3_BO_3_ (pH 9) buffer and a NaHCO_3_ buffer. It is evident that the current density at 1.23 V in the H_3_BO_3_ electrolyte is 1.9 mA/cm^2^, reflecting a 65% reduction compared to the BVO in the NaHCO_3_ electrolyte. This disparity may be attributed to the challenge of effectively adsorbing BA onto the electrode surface for a catalytic reaction in the H_3_BO_3_ electrolyte. Table S1 outlines the FE of BA oxidation in various electrolytes, with the FE of BAD formation in the H_3_BO_3_ electrolyte registering at a mere 1.5%.

Because the literature on metal surface catalysis generally uses strong alkalinity [32], the LSV outcomes of BVO in a 0.1 M KOH electrolyte (pH 13.5) versus the NaHCO_3_ electrolyte reveal a photocurrent density of 3 mA/cm^2^ at 1.23 V, indicating a 45% decline compared to the NaHCO_3_ electrolyte. Notably, the peak current density occurs concurrently with the current’s rise, leading to rapid recombination of photogenerated carriers. While bases are commonly utilized mediums for electrocatalysis on metal surfaces and are deemed beneficial for hydroxyl oxidation, the mechanism primarily involves oxygen adsorption on the metal surface to facilitate the oxidation reaction, differing substantially from oxide surface interactions. Apart from adsorption at the active site of the REDOX reaction, oxide surfaces engage in charge transfer and intermediate formation to enhance catalytic efficacy. Moreover, SEM (Figure S5) observations of the BVO morphology indicate that corrosion levels in H_3_BO_3_ and NaHCO_3_ electrolytes are less severe than those in the KOH electrolyte, suggesting accelerated BVO dissolution in the alkaline electrolyte, resulting in a significantly decreased BVO utilization efficiency.

Our objective was to analyze the charge transport behavior in three distinct electrolytes by implementing electrochemical impedance spectroscopy (EIS) in conjunction with the relaxation time distribution (DRT) tool [33], which effectively disentangles overlapping polarization processes within EIS. Figure 3b delineates the electron transfer dynamics of oxidized BA at 0.6 V versus the reversible hydrogen electrode (RHE) in three electrolytes through BVO. The low-frequency peak corresponds to the BVO–electrolyte interface, the medium-frequency peak signifies the charge transfer of BA oxidized by BVO, and the high-frequency peak relates to the polarization process of BVO itself. The results showcase that, in the NaHCO_3_ electrolyte, the mid-frequency peak intensity is the lowest, indicative of a prolonged light-induced charge separation duration within this system. This leads to the effective separation of electron–hole pairs and a heightened charge transfer rate. Conversely, the H_3_BO_3_ system reveals ineffective charge separation within BVO, accompanied by elevated charge transfer resistance.

Furthermore, the semi-arc observed at low frequencies in the Nyquist plot of Figure 3c provides key insights into the charge transfer resistance characteristics. Notably, the smallest radius of the semi-arc observed in the NaHCO_3_ test directly demonstrates that BVO exhibits a higher charge injection rate in this specific system compared to the other two electrolytes. Figure 3d present additional scrutiny through Bode plots, portraying BVO testing at 0.6 V versus RHE in the three systems, the frequency magnitude that are capable of elucidating the time domain characteristics of the kinetic process. Across all systems, the most significant phase shift is observed in the low-frequency domain, pointing towards charge transfer restriction by dynamics. Notably, the phase angle of the BVO diminishes in the MeCN electrolyte containing NaHCO_3_, signifying enhanced interface conductivity and improved charge transfer kinetics [34,35]. Within this system, the frequency response transition is smoother, inducing a more stable and uniform response.

**Figure 4:**
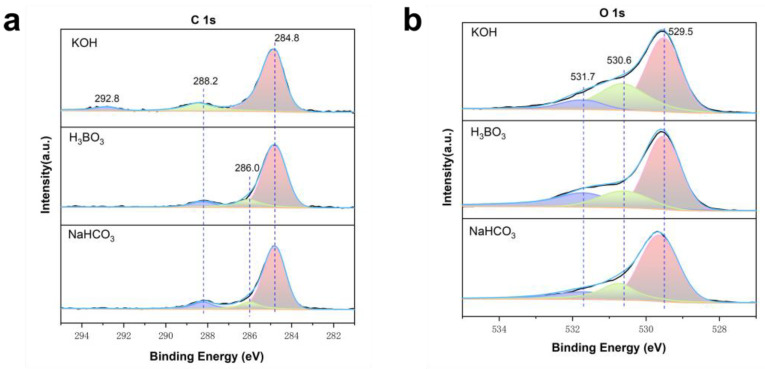
(**a**) C 1s of XPS spectrum after 30 min CA testing of BVO in 0.1 M KOH, 0.5 M H_3_BO_3_, and MeCN electrolyte containing 0.5 M NaHCO_3_ and (**b**) O 1s of XPS spectrum after 30 min CA testing of BVO in 0.1 M KOH, 0.5 M H_3_BO_3_, and MeCN electrolyte containing 0.5 M NaHCO_3_.

We conducted an analysis to determine if carbonate substances were generated on the surface of BVO following testing in the NaHCO_3_ electrolyte and observed any notable alterations in surface elements after testing in the remaining two electrolytes. X-ray photoelectron spectroscopy (XPS) was employed to elucidate changes in the elemental chemical composition and oxidation state of BVO after a 30 min reaction with various electrolytes. Figure 4a illustrates the high-resolution energy spectrum of C 1s, where peaks at 284.8 eV correspond to C-C bonds, peaks at 286 eV relate to C-O-C bonds, and peaks slightly above 288 eV signify O-C=O or -C-OH bonds. The peak at 286 eV in the BVO may be attributed to the reaction with organic matter. The C 1s peak of the BVO after reacting with the NaHCO_3_ electrolyte was higher than that of the pristine BVO and registered at 288.2 eV after the reaction with the H_3_BO_3_ electrolyte, indicating the presence of carbonate material [36,37]. Following the reaction with the KOH electrolyte, the BVO displayed a peak at 292.8 eV, representing potassium [38].

In Figure 4b, the high-resolution energy spectrum of O 1s showcases lattice oxygen at 529.5 eV, surface oxygen at 530.6 eV, and non-metallic oxide at 531.7 eV. Post the reaction of BVO in the NaHCO_3_ electrolyte, the surface peak area at 529.5 eV increased, signifying the deposition of carbonate material. The heightened peak intensity of the BVO at 530.6 eV after reacting in the KOH electrolyte results from oxygen adsorption on the surface. The peak intensity of the BVO at 531.7 eV following the reaction in the NaHCO_3_ electrolyte is lower than under the other conditions, consistent with previous findings [36]. The element ratio of surface composition in Table S2 falls within an acceptable range. There is minimal disparity between the V 2p and Bi 4f high-resolution energy spectrum peaks, indicating that the electron density surrounding the BVO surface elements remains relatively stable (Figure S6).

### 2.4. Influence of Solvent on Electrolyte System

We opted for MeCN as a solvent instead of pure water based on research by Li et al. [19], confirming the enhanced photostability of oxidized BA in MeCN media. This finding underscores the suitability of BA oxidation in non-aqueous environments. Furthermore, MeCN serves as a potent solvent that promotes the oxidation of BA, facilitating its dissolution along with the resultant products. MeCN’s robust light-absorption characteristics foster favorable conditions for photocatalytic reactions. Our study delved into and compared the distinct effects of MeCN and pure water as solvents.

**Figure 5:**
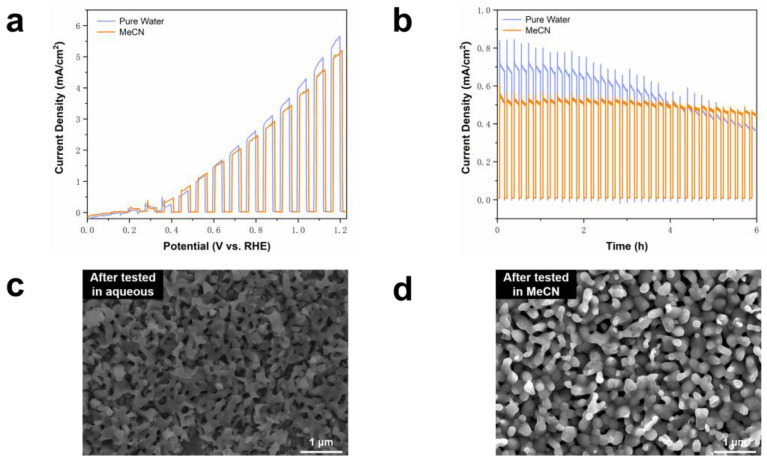
(**a**) BVO test results of LSV in aqueous solution buffer and MeCN buffer containing 0.5 M NaHCO_3_ at pH 9; (**b**) BVO test results of 6 h CA in aqueous solution buffer and MeCN buffer containing 0.5 M NaHCO_3_ at pH 9; (**c**) BVO morphology after CA test in aqueous buffer containing NaHCO_3_; (**d**) BVO morphology after CA test in MeCN buffer containing NaHCO_3_.

Figure 5a illustrates the outcomes of BVO testing through LSV in MeCN solution with NaHCO_3_ as the electrolyte, alongside water solution with NaHCO_3_ as the electrolyte under identical conditions. The results indicate a decrease in photocurrent density in the MeCN system, which can potentially be attributed to reduced light energy conversion efficiency following the introduction of MeCN. The HPLC data in Table S1 reveal a higher FE in oxidizing BA to BAD in the MeCN electrolyte compared to the water electrolyte. This disparity is attributed to the stronger polarity and enhanced solubility of BA in the aprotic solvent MeCN. Moreover, BAD attains optimum dispersion in MeCN solution. 

In Figure 5b, the enhanced photostability of BVO in the MeCN system after 6 h surpasses that in the water system, mainly due to the small solubility of inorganic matter in organic solvent. Notably, the MeCN system decelerates the corrosion process of BVO in water. Observations from Figure 5c,d regarding the morphology of BVO indicate significantly reduced corrosion in the MeCN electrolyte compared to the water electrolyte. 

**Figure 6:**
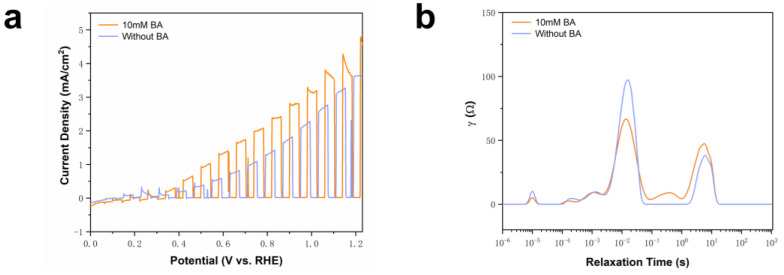
(**a**) BVO test results of LSV adding BA and no BA in 0.5 M NaHCO_3_ MeCN buffer of pH 9; (**b**) BVO test results of EIS at 0.6 V vs. RHE adding BA and no BA in 0.5 M NaHCO_3_ MeCN buffer of pH 9.

Figure 6a demonstrates a 30% increase in the current of BVO when BA is introduced into the MeCN electrolyte with NaHCO_3_, suggesting the active oxidation of BA. In Figure 6b, the distribution of relaxation times (DRT) tool was employed within EIS to gain insights into the charge transfer properties. The result reveals a decrease in the peak intensity at a medium frequency following the introduction of BA, resulting in reduced resistance to charge transfer at the BVO photoanode/electrolyte interface [39].

### 2.5. HCO_3_^−^ Mediated the Oxidation of Benzyl Alcohol by BVO

When organic matter undergoes oxidation in a bicarbonate system, there is significant adsorption of bicarbonate on the surface of BVO. This interaction results in the generation of photogenerated holes between HCO_3_^−^ and BVO, as well as at the catalyst/electrolyte interface, thereby enhancing the charge transfer reaction. This leads to the formation of carbonate free radicals (·CO_3_^−^). Acting as a single electron catalyst, the carbonate free radical exhibits a rapid reaction rate with the BA, a process that is well-documented [40]. We are exploring the hypothesis that the pH value of the electrolyte, as well as the concentration of bicarbonate, could impact the results. Specifically, we are investigating whether higher concentrations of bicarbonate can generate more carbonate radicals, potentially increasing the efficiency of BA oxidation. The concentrations of carbonate and bicarbonate are closely linked to the pH value. Therefore, our study delves into the impact of the NaHCO_3_ concentration and pH on the oxidation of BA by BVO.

**Figure 7:**
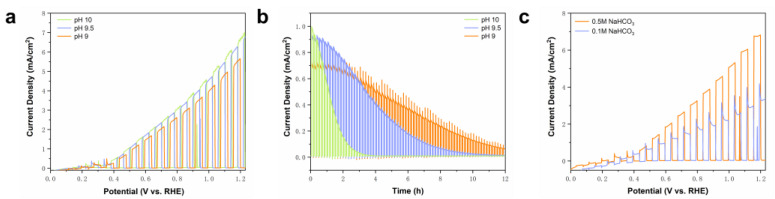
(**a**) LSV of BVO in aqueous electrolyte containing NaHCO_3_ in pH 9, pH 9.5, and pH 10; (**b**) CA of BVO at 0.6 V vs. RHE in aqueous electrolyte containing NaHCO_3_ in pH 9, pH 9.5, and pH 10 for 12 h; (**c**) LSV of BVO in aqueous electrolyte containing 0.1 M and 0.5 M NaHCO_3_ (pH 9).

In Figure 7a, the results of LSV in aqueous solutions containing 0.5 M NaHCO_3_ at pH 9, pH 9.5, and pH 10 with BVO as the electrolyte are presented. The findings indicate that the current density of BVO at 1.23 V reaches 7 mA/cm^2^ in the pH 10 system, while the photocurrent density at 1.23 V in the pH 9 system is 5.9 mA/cm^2^. The photocurrent typically correlates with the electrolyte concentration and the type of anion present. Notably, the enhanced charge transfer reaction at the photogenerated holes and the photoanode/electrolyte interface facilitates the formation of oxidizing species such as carbonate free radicals, consequently boosting the photocurrent.

Despite the higher photocurrent density observed in the pH 10 system, the photostability results in Figure 7b, recorded at a voltage of 0.5V over 12 h, indicate that the pH 9 electrolyte exhibits greater stability. Additionally, the BVO displayed faster dissolution in the more alkaline environment. The HPLC test outcomes in Table S1 reveal that 23% of the FE in the pH 9 electrolyte facilitated the oxidation of BA to BAD, while the pH 10 electrolyte experienced dissolution without generating BAD. The alkaline nature of the pH 10 condition is unfavorable for the conversion of BA to BAD. Considering the correlation between the concentration of carbonate compounds and the pH levels (Figure S7), the HCO_3_^−^ concentration is higher in the pH 9 system, while it is significantly reduced in the pH 10 system, leading to a decrease in carbonate free radicals. Consequently, the oxidation efficiency of the BA is compromised, underscoring the significance of bicarbonate in the BA oxidation process. 

In Figure 7c, we examined the outcomes of the BVO testing through LSV in aqueous solutions containing 0.1 M and 0.5 M NaHCO_3_ at pH 9. The results indicate that the photocurrent density increases with HCO_3_^−^ concentration, confirming the pivotal role played by HCO_3_^−^ in the oxidation of BA by BVO.

We endeavored to introduce ascorbic acid into the electrolyte containing NaHCO_3_, as it has the capability to eliminate carbonate free radicals (Figure S8) [41,42]. In Table S1, the HPLC findings after 2 h revealed that the BA failed to transform into BAD in the NaHCO_3_ electrolyte supplemented with ascorbic acid. This outcome underscores the pivotal role played by carbonate free radicals in determining the oxidation of BA by BVO within the NaHCO_3_ electrolyte.

## 3. Discussion

**Figure 8:**
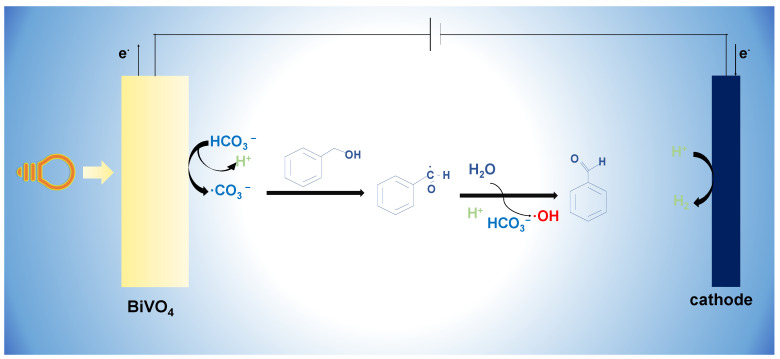
Action mechanism diagram of bicarbonate.

As depicted in Figure 8, the irradiation of BVO with light results in the selective adsorption of BA on the BVO surface. Subsequently, HCO_3_^−^ undergoes oxidation to generate the carbonate free radical ·CO_3_^−^, which further reacts by attacking the C-O bond of the hydroxyl group in the BA. This process leads to the formation of a carbon central free radical, which then hydrolyzes and combines with the hydroxyl radical. The activation of the C-O bond culminates in the production of BAD [23]. Concurrently, protons are reduced to hydrogen at the cathode. Notably, the carbonate free radical exhibits a higher propensity to bind to the target compared to the hydroxyl free radical.

Our study demonstrates the advantageous role of bicarbonate as an electrolyte in enhancing photoelectrocatalytic efficiency, laying the groundwork for catalytic reactions or organic matter degradation facilitated by bicarbonate. We have made enhancements to stabilize the bicarbonate electrolyte system and effectively mitigate the impact of BVO photocorrosion under alkaline conditions by introducing a non-aqueous medium and saturated V^5+^. However, there remains a need to enhance the FE in oxidizing BA. In the future, we can improve the selectivity of BVO to oxidize benzyl alcohol by surface modification, such as loading a cocatalyst or constructing a heterojunction [43,44,45]. After all, the improvement of BVO itself is also the most direct way.

## 4. Materials and Methods

### 4.1. All the Chemical Reagents and Manufacturers in the Experiment

Bismuth nitrate pentahydrate (Bi(NO_3_)_3_·5H_2_O, 99%), potassium iodide (KI, 99%), and p-benzoquinone (C_6_H_4_O_2_, 99%) for the preparation of BVO in the experiment were from Aladdin (Shanghai, China). Vanadium acetylacetonate (C_10_H_14_O_5_V, 99%) and vanadium pentoxide (V_2_O_5_, 99%) were from MACKLIN (Shanghai, China). The solvent used was nitric acid (HNO_3_) from MACKLIN. Dimethyl sulfoxide (C_2_H_6_OS) was from Aladdin. Acetonitrile (CH_3_CN) was obtained from Aladdin, sodium bicarbonate (NaHCO_3_, ≥99.8%) and sodium carbonate (Na_2_CO_3_, ≥99.8%) were obtained from MACKLIN. Potassium hydroxide (KOH, 90%), sodium hydroxide, sodium sulfite, and boric acid (H_3_BO_3_, 99.99%) come from MACKLIN. Benzyl alcohol (C_6_H_5_CH_2_OH, >99.0%) and benzaldehyde (C_7_H_6_O, >99.0%) are sourced from Aladdin.

### 4.2. Preparation Method of BVO

BVO was prepared by electrodeposition, and the electrolyte was prepared with 1 M dilute nitric acid to adjust the pH of potassium iodide solution to 1.7. Bismuth nitrate pentahydrate was added and stirred until dissolved, and then para-benzoquinone was dissolved in ethanol. The two dissolved slowly and BiOI was obtained by deposition in a three-electrode system. Vanadyl acetonate was dissolved in DMSO, sprayed on the surface of BiOI, and then heated in Muffle furnace at 450 °C for 2 h to obtain BVO samples. Excessive vanadium was removed by soaking in aqueous NaOH solution.

### 4.3. PEC Test Method

PEC tests are performed in a three-electrode system (unless otherwise indicated), with BVO photoanode as the working electrode, Pt wire as the opposing electrode, and Ag/AgCl as the reference electrode. Electrochemical workstation is CHI550E from Chenhua (Shanghai, China). Using LED 455 nm blue light as a light source, the light intensity is 39 mW/cm^2^.

### 4.4. Material Characterization Equipment

The morphologies of the samples were obtained by Hitachi S4800 scanning electron microscope from Science (Suzhou, China). Ultraviolet-visible absorption spectra were recorded on the Perkin-Elmer Lambda 950 ultraviolet-visible near-infrared spectrometer from PERKINELMER (Waltham, MA, USA). X-ray photoelectron spectroscopy (XPS) measurements were made on Thermo Scientific K-Alpha and Al Ka (1486.6 eV) instruments from Thermo Scientific (Waltham, MA, USA). The binding energy was corrected with C 1s of the indeterminate carbon 284.8 eV.

## 5. Conclusions

We conducted a study to examine the impact of different electrolyte systems on the catalysis of BA by BVO. The incorporation of a non-aqueous medium and saturated V^5+^ serves to deter the photocorrosion of bismuth vanadate and bolster water oxidation kinetics. While bicarbonate, as an electrolyte, demonstrates the ability to catalyze the oxidation of BA to BAD, its Faraday efficiency has not shown significant enhancement. To optimize the efficiency of bismuth vanadate in catalyzing BA, the selection of an appropriate electrolyte system stands out as a simpler and safer approach.

## Data Availability

Data are contained within the article and Supplementary Materials.

## Supplementary Materials

The following supporting information can be downloaded at: https://www.mdpi.com/article/10.3390/molecules29071554/s1, Figure S1. BVO activated LSV in boric acid buffer (pH 9) containing 0.2 M Na_2_SO_3_. Figure S2. BVO tested LSV added saturated V^5+^ and without V^5+^ to the MeCN electrolyte (pH 9) containing 0.5 M NaHCO_3_. Figure S3. Comparison of thickness of BVO with saturated V^5+^ and without V^5+^ in MeCN electrolyte (pH 9) containing 0.5 M NaHCO_3_ was observed by naked eye. Figure S4. Schematic diagram of assembling a flow pool. Figure S5. BVO test SEM after 6h testing in 0.5 M H_3_BO_3_, 0.1 M KOH, and MeCN containing 0.5 M NaHCO_3_. Figure S6. (a) Bi 4f of XPS spectrum after 30 min testing of BVO in 0.1 M KOH, 0.5 M H_3_BO_3_ and MeCN electrolyte containing 0.5 M NaHCO_3_ (b) V 2p of XPS spectrum after 30 min testing of BVO in 0.1 M KOH, 0.5 M H_3_BO_3_ and MeCN electrolyte containing 0.5 M NaHCO_3_. Figure S7. Carbonic acid species as a function of pH at 25 °C for total ion activity coefficients equal to unity [46]. Figure S8. BVO test results of LSV added ascorbic acid and without ascorbic to MeCN electrolyte pH 9 containing 0.5 M NaHCO_3_. Figure S9. (a) HPLC results of BVO tests 6 h for 0.5 M NaHCO_3_ MeCN electrolytes with saturated V^5+^ and without V^5+^ (b) HPLC results of BVO tests 12 h for 0.5 M H_3_BO_3_, 0.1 M KOH, and MeCN containing 0.5 M NaHCO_3_ (c) HPLC results of BVO test 12 h in aqueous electrolytes containing NaHCO_3_ at pH 9, pH 9.5 and pH 10 (d) HPLC results of BVO test 12 h for 0.5 M NaHCO_3_ MeCN electrolytes with ascorbic acid and without ascorbic. Table S1. Results of HPLC tests in different systems. Table S2. XPS surface composition ratio of elements.
