# Supplementary material for: Stabilizing BiVO4 Photoanode in Bicarbonate Electrolyte for Efficient Photoelectrocatalytic Alcohol Oxidation
Source: Molecules. 2024 Mar 30;29(7):1554. doi: 10.3390/molecules29071554 (PMC11013117; doi:10.3390/molecules29071554)
Supplement: Supplementary file 1 [file molecules-29-01554-s001.zip › molecules-2933326-supplementary.pdf]

Supporting information

Stabilizing BiVO4 photoanode in bicarbonate electrolyte for efficient photoelectrocatalytic alcohol oxidation

Haorui Gong [1], Sai An [1], Yongbo Kuang [2,3] *, Weilong Qin[2], and Deyu Liu [2,] *

[1] School of Materials Science and Chemical Engineering, Ningbo University, Ningbo, Zhejiang 315211, China.; gonghaorui@nimte.ac.cn (H.G.)
[2] Key Laboratory of Advanced Fuel Cells and Electrolyzers Technology of Zhejiang Province, Ningbo Institute of Materials Technology and Engineering, Chinese Academy of Sciences, 1219 Zhongguan West Road, Ningbo, Zhejiang 315201, China; qinweilong@nimte.ac.cn (W.Q)
[3] Center of Materials Science and Optoelectronics Engineering, University of Chinese Academy of Sciences, Beijing 100000, China; kuangyongbo@nimte.ac.cn (Y.K)
* Correspondence: kuangyongbo@nimte.ac.cn (Y.K); dyliu.xiamen@gmail.com (D.L.)

1.Calculations of Faradaic efficiency

Faradaic efficiency (%) = $\frac{e\times n1\times N}{Q/n2}$

Where e is the number of holes required to oxidize one BA to BAD(e=2), n1 is the amount of substance that produces BAD, N is Avogadro's constant($N=6.02\times10^{23}$), Q is the quantity of electric charge, and n2 is the elementary charge($e=1.602\times10^{-19}$).

2.Figure S1-S9

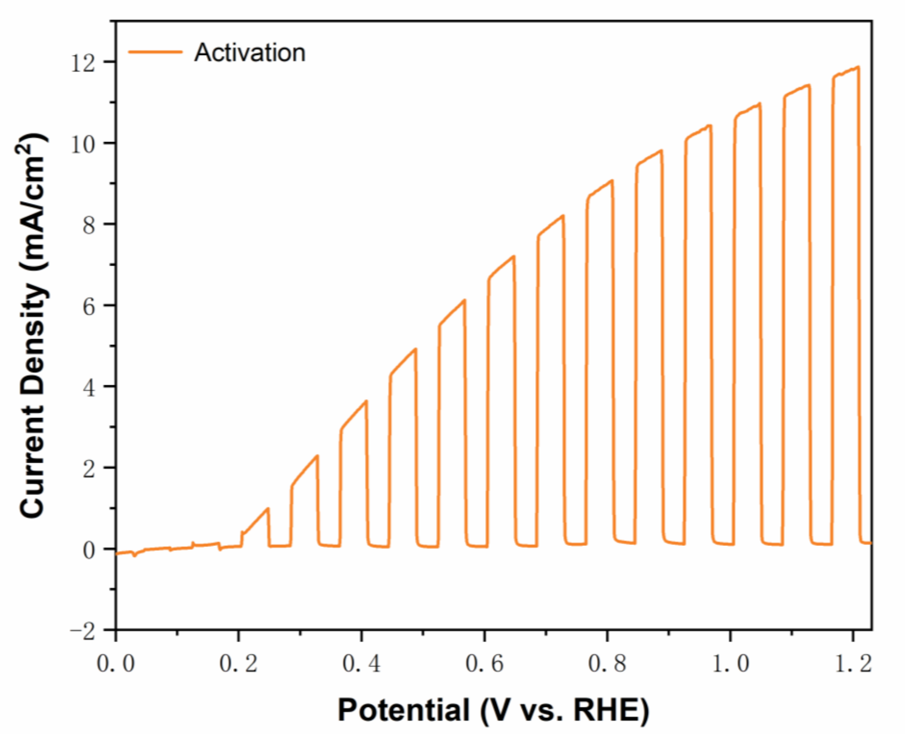

Figure S1. BVO activated LSV in boric acid buffer (pH 9) containing 0.2 M Na_2SO_3

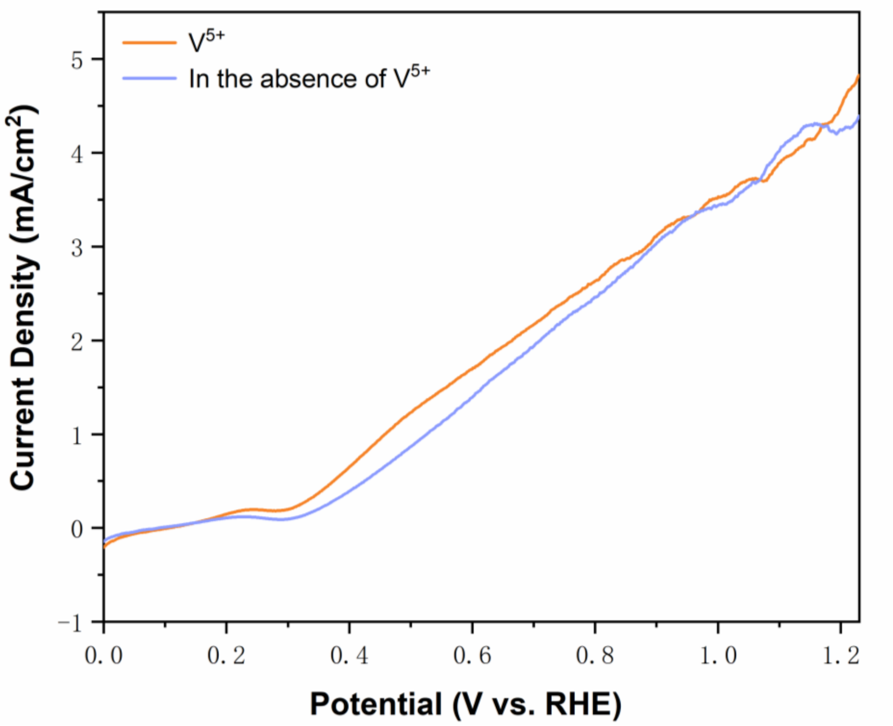

Figure S2. BVO tested LSV added saturated V^{5+} and without V^{5+} to the MeCN electrolyte (pH 9) containing 0.5 M $NaHCO_3$

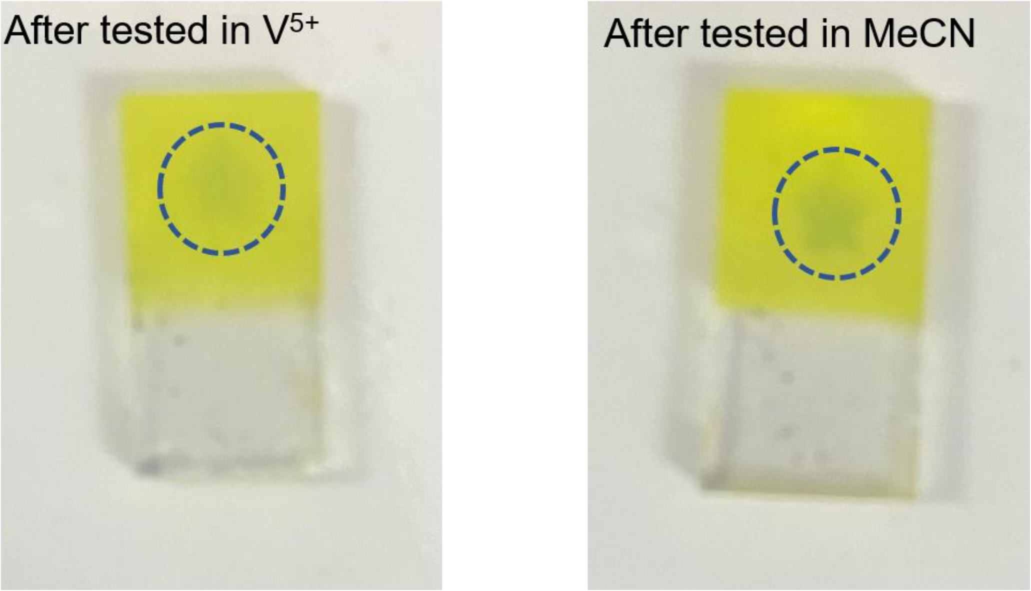

Figure S3. Comparison of thickness of BVO with saturated V^{5+} and without V^{5+} in MeCN electrolyte (pH 9) containing 0.5 M $NaHCO_3$ was observed by naked eye

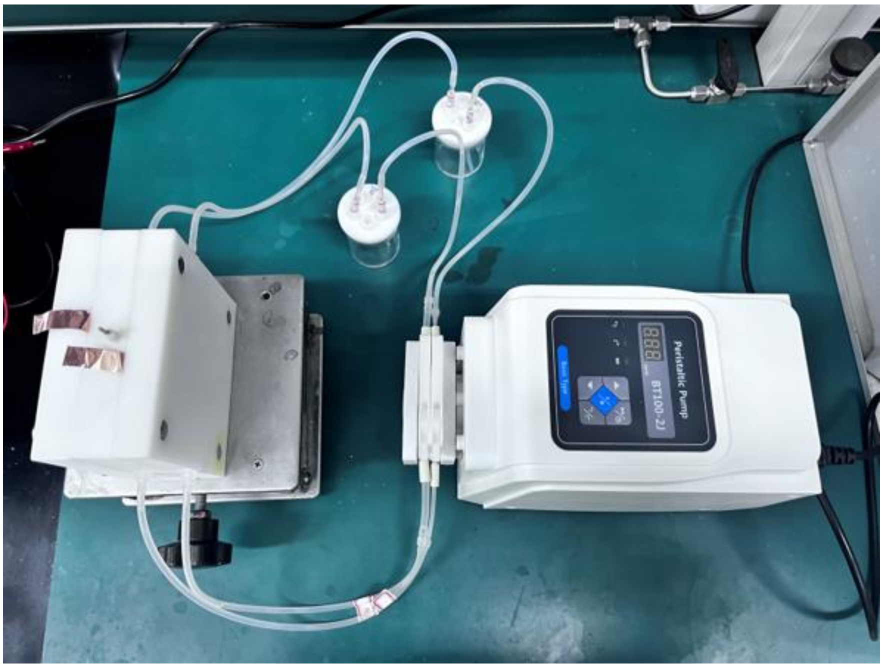

Figure S4. Schematic diagram of assembling a flow pool

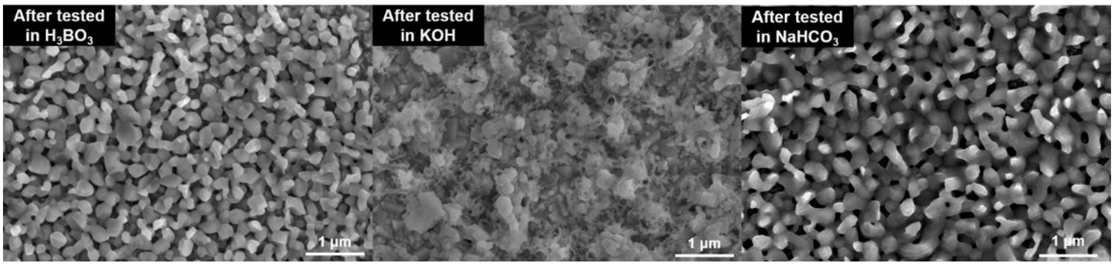

Figure S5. BVO test SEM after 6h testing in 0.5 M H_3BO_3, 0.1M KOH, and MeCN containing 0.5 M $NaHCO_3$

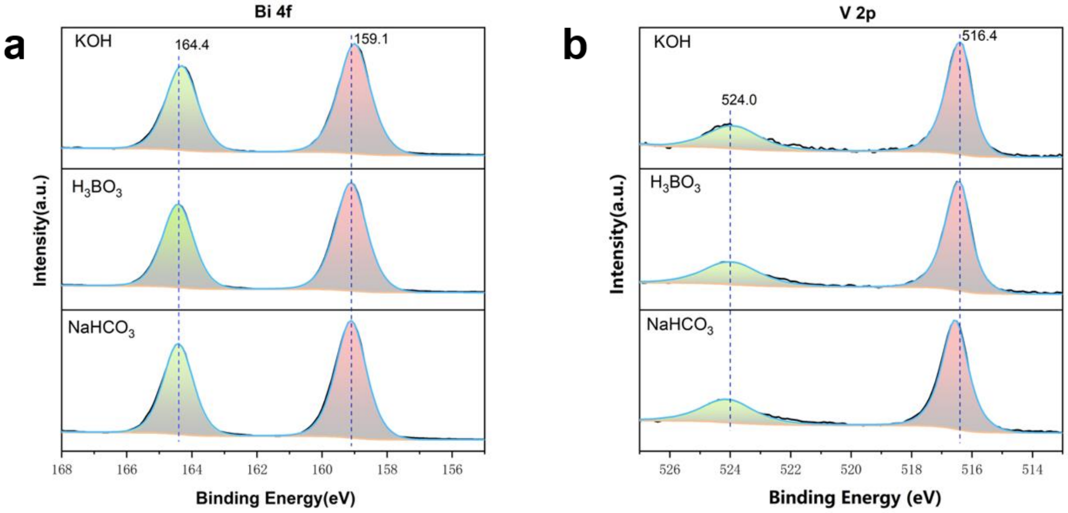

Figure S6. (a) Bi 4f of XPS spectrum after 30 min testing of BVO in 0.1 M KOH, 0.5 M H_3BO_3 and MeCN electrolyte containing 0.5 M $NaHCO_3$ (b) V 2p of XPS spectrum after 30 min testing of BVO in 0.1 M KOH, 0.5 M H_3BO_3 and MeCN electrolyte containing 0.5 M $NaHCO_3$

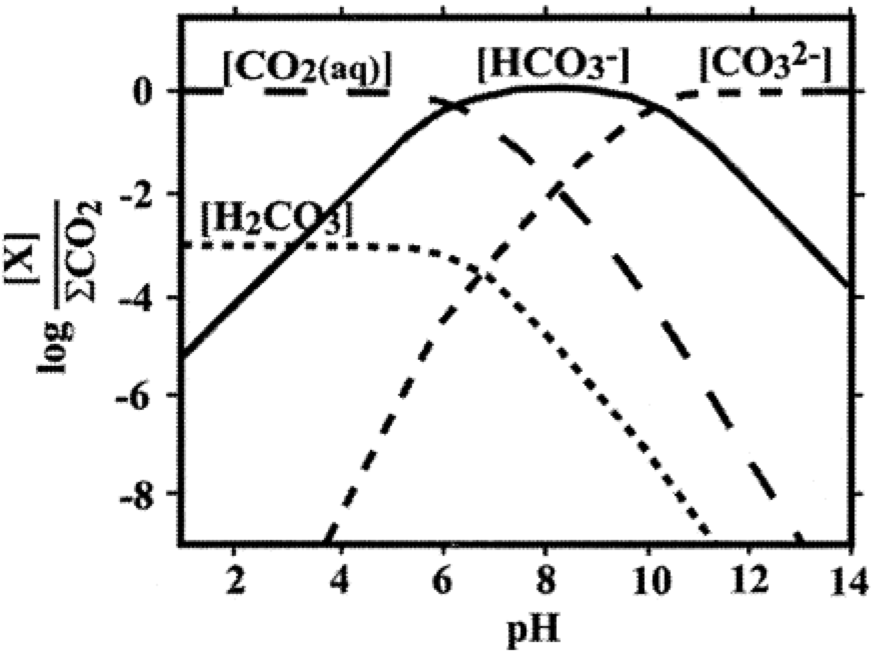

Figure S7. Carbonic acid species as a function of pH at 25 °C for total ion activity coefficients equal to unity[1].

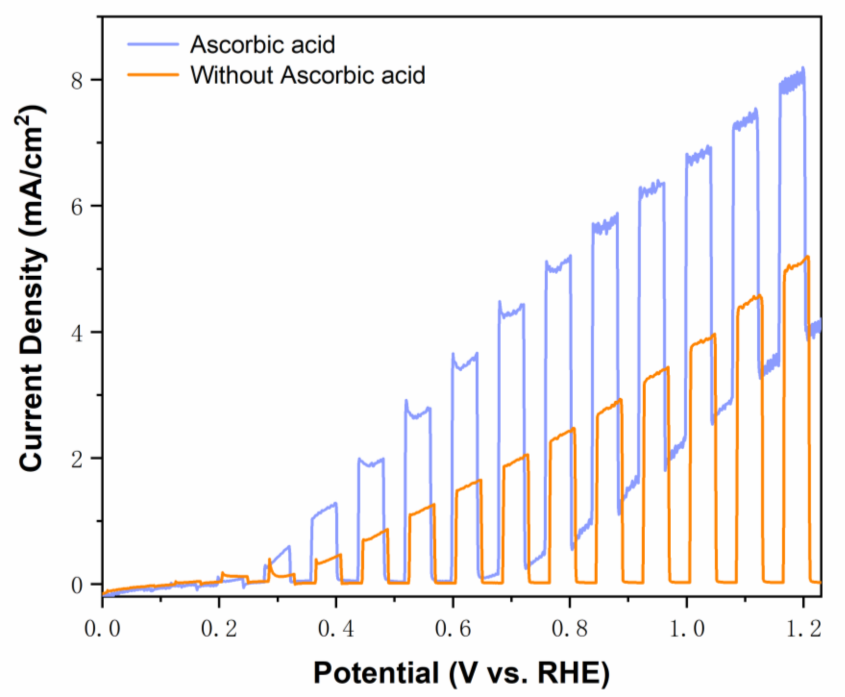

Figure S8. BVO test results of LSV added ascorbic acid and without ascorbic to MeCN electrolyte pH 9 containing 0.5 M $NaHCO_3$

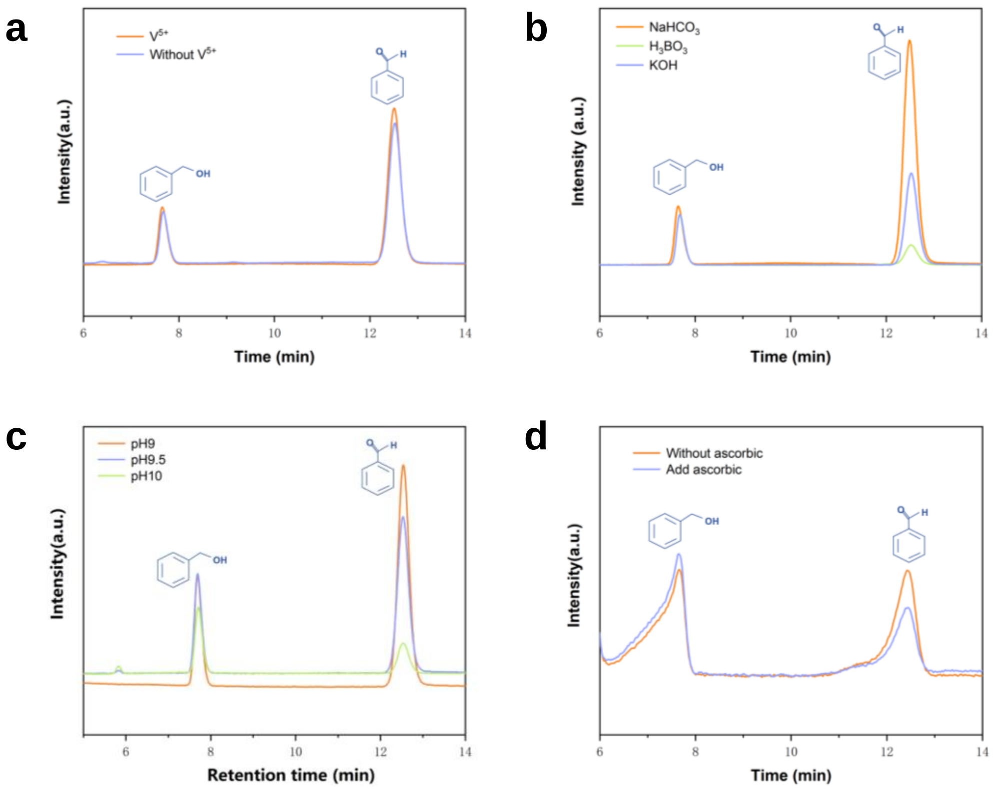

Figure S9. (a) HPLC results of BVO tests 6 h for 0.5 M $NaHCO_3$ MeCN electrolytes with saturated V^{5+} and without V^{5+}(b) HPLC results of BVO tests 12 h for 0.5 M H_3BO_3, 0.1 M KOH, and MeCN containing 0.5 M $NaHCO_3$ (c) HPLC results of BVO test 12 h in aqueous electrolytes containing $NaHCO_3$ at pH 9, pH 9.5 and pH 10(d) HPLC results of BVO test 12 h for 0.5 M $NaHCO_3$ MeCN electrolytes with ascorbic acid and without ascorbic.

3.Table S1-S2

Table S1. Results of HPLC tests in different systems

Electrolyte	Time(h)	Faraday efficiency(%)
0.5 M $NaHCO_3$ in MeCN add V^{5+} (pH 9)	6 h	25.9±5
0.5 M $NaHCO_3$ in MeCN (pH 9)	6 h	20.1±5
0.5 M H_3BO_3 (pH 9)	6 h	1.5±5
0.1 M KOH (pH 13.5)	6 h	12.7±5
0.5 M $NaHCO_3$ in aqueous (pH 9)	12 h	23±5
0.5 M $NaHCO_3$ in aqueous (pH 9. 5)	12 h	20±5
0.5 M $NaHCO_3$ in aqueous (pH 10)	12 h	0
0.5 M $NaHCO_3$ in MeCN (pH 9)	2 h	10±5
0.5 M $NaHCO_3$ in MeCN add ascorbic (pH 9)	2 h	0

Table S2. XPS surface composition ratio of elements

Element	KOH[1]	H_3BO_3[2]	$NaHCO_3$[3]
Bi	9.05	8.04	8.59
V	15.44	18.82	20.14
O	35.53	37.47	38.47
C	38.97	35.65	35.56

[1] After 30 min testing of BVO in 0.1 M KOH, [2] After 30 min testing of BVO in 0.5 M H_3BO_3, [3] After 30min testing of BVO in 0.5 M $NaHCO_3$

4.References

1. Beck, W.C.; Grossman, E.L.; Morse, J.W. Experimental Studies of Oxygen Isotope Fractionation in the Carbonic Acid System at 15°, 25°, and 40°C. *Geochim. Cosmochim. Acta* **2005**, *69*, 3493–3503, doi:10.1016/j.gca.2005.02.003.
